# Use of laboratory testing for prediction of postoperative bleeding volume in cardiovascular surgery

**DOI:** 10.1186/s12959-021-00324-4

**Published:** 2021-10-09

**Authors:** Yoshie Kawahara, Kohei Ohtsuka, Kimine Tanaka, Mayumi Yamanaka, Hiroyuki Kamiya, Takayuki Kunisawa, Satoshi Fujii

**Affiliations:** 1 Department of Medical Laboratory and Blood Center, Asahikawa Medical University Hospital, Midorigaoka-Higashi 2-1-1-1, Asahikawa, 078-8510 Japan; 2 Present address: Japanese Red Cross Hokkaido Block Blood Center, Sapporo, Japan; 3 Department of Cardiac Surgery, Asahikawa Medical University, Asahikawa, Japan; 4 Department of Anesthesiology, Asahikawa Medical University, Asahikawa, Japan

**Keywords:** Cardiovascular surgery, Diluted coagulopathy, Hemorrhage, Laboratory tests

## Abstract

**Background:**

Coagulopathy and following massive bleeding are complications of cardiovascular surgery, particularly occurring after procedures requiring prolonged cardiopulmonary bypass (CPB). Reliable and rapid tests for coagulopathy are desirable for guiding transfusion. Measuring multiple coagulation parameters may prove useful. The purpose of this study is to determine the laboratory parameters predicting massive bleeding.

**Methods:**

In a prospectively collected cohort of 48 patients undergoing cardiovascular surgery, markers of coagulation and fibrinolysis were measured using automated analyzer and their correlations with bleeding volume were determined.

**Results:**

Operation time was 318 (107–654) min. CPB time was 181 (58–501) min. Bleeding volume during surgery was 2269 (174–10,607) ml. Number of transfusion units during surgery were packed red blood cells 12 (0–30) units, fresh frozen plasma 12 (0–44) units, platelets 20 (0–60) units and intraoperative autologous blood collection 669 (0–4439) ml. Post-surgery activities of coagulation factors II (FII), FV, FVII, FVIII, FIX, FX, FXI and FXII were decreased. Values of fibrinogen, antithrombin, α2 plasmin inhibitor (α2PI) and FXIII were also decreased. Values of thrombin-antithrombin complex (TAT) were increased. Values of FII, FIX, FXI and α2PI before surgery were negatively correlated with bleeding volume (FII, *r =* − 0.506: FIX, *r =* − 0.504: FXI, *r =* − 0.580; α2PI, *r =* − 0.418). Level of FIX after surgery was negatively correlated with bleeding volume (*r =* − 0.445) and level of TAT after surgery was positively correlated with bleeding volume (*r =* 0.443).

**Conclusions:**

These results suggest that several clinical and routine laboratory parameters of coagulation were individually associated with bleeding volume during cardiovascular surgery. Determining the patterns of coagulopathy may potentially help guide transfusion during cardiovascular surgery.

## Background

Coagulopathy and massive bleeding are severe complications of cardiovascular surgery, particularly occurring after procedures requiring prolonged cardiopulmonary bypass (CPB) [1, 2]. During surgery massive transfusion of blood products may become necessary. Transfusion practices may vary among different countries [3]. To predict and prevent massive bleeding thoracic surgeons and cardiovascular anesthesiologists have made substantial efforts [4]. Reliable and predictive laboratory tests on coagulation function may help guide clinicians to appropriate transfusion and may decrease variabilities in transfusion practices. Appropriate coagulation parameters would help to predict bleeding after cardiovascular surgery. Such knowledge could potentially enable clinicians to effectively treat coagulopathy after surgery requiring CPB. However, risk scores for blood transfusion [5] and hemostatic laboratory tests have not been fully evaluated for hemorrhagic risk prediction in Asian patients. The purpose of this study is to determine the markers predicting massive bleeding in Japanese patients. Markers of coagulation and fibrinolysis were measured during cardiovascular surgery and their correlations with bleeding volume were determined.

## Methods

### Study design and subjects

In a prospectively collected cohort of patients undergoing cardiovascular surgery at Asahikawa Medical University Hospital (Asahikawa, Japan) from November 2015 to March 2016, we examined the association between coagulation parameters and bleeding volume. The characteristics of the subjects are shown in Table 1. This study was approved by the institutional review board (approval number 14127). Informed consent was obtained from individual patient for the use of blood normally discarded after routine laboratory coagulation testing and medical records. Clinicians caring for patients were not provided with assay results. Institutional clinical or transfusion practices were unchanged during the study period. Heparin (300 units/kg body weight) was used in each case during CPB and great care was taken so that blood samples for analysis would avoid heparin contamination.

**Table 1:** Demographic and operative patient characteristics

Characteristic	Median (IQR) or n (%)	
Age (years)	69	(39–85)
Gender		
Male	29	(60%)
Female	19	(40%)
Race		
Asian	48	(100%)
Operative Data		
Operation time (min)	318	(107–654)
CPB time (min)	181	(58–501)
Bleeding volume (ml)	2269	(174–10,607)
Blood Transfusion		
RBC (units)	12	(0–30)
FFP (units)	12	(0–44)
PC (units)	20	(0–60)
Intraoperative autologous		
Blood collection (ml)	669	(0–4439)
Type of operation		
CABG only	6	(13%)
Valve surgery only	22	(46%)
CABG and valve surgery	3	(6%)
CABG and aortic surgery	11	(23%)
Valve and aortic surgery	2	(4%)
Other procedures	4	(8%)

*CPB* cardiopulmonary bypass, *RBC* red blood cell, *FFP* fresh frozen plasma, *PC* platelet concentrate, *CABG* coronary artery bypass grafting

### Data and sample collection

Patients’ medical records were reviewed for surgical procedures, general laboratory test results and blood product transfusions. One milliliter of citrated whole blood was obtained from specimens collected as part of routine clinical care. For each case blood was obtained at 2 points. Time of sampling included (point 1) pre-CPB after induction of anesthesia, or at the time immediately before surgery in patients without CPB, and (point 2) post-CPB, or at the time right after surgery procedure in patients without CPB.

### Analysis

After centrifuge blood samples were stored below − 30 °C. PT, APTT and fibrinogen (Fib) were measured with automated analyzer (coagulation one-stage assay, Coapresta2000, Sekisui Medical Company, Tokyo, Japan). Activities of coagulation factor II (FII), FV, FVII, FVIII, FIX, FX, FXI and FXII were measured with Coapresta3000 (coagulation one-stage assay, Sekisui Medical). α2 Plasmin inhibitor (α2PI, synthetic substrate assay), antithrombin (AT, synthetic substrate assay), thrombin-antithrombin complex (TAT, chemiluminescent enzyme immunoassay) and FXIII activity (synthetic substrate assay) were measured using automated analyzer (STACIA, LSI Medience Corporation, Tokyo, Japan).

### Statistical methods

Data are reported as box plot with lower extreme, lower quartile, median, upper quartile and upper extreme values. Student or Mann-Whitney rank sum tests for quantitative variables were used to determine differences in unadjusted preoperative and postoperative characteristics. A *p* value less than 0.05 was considered as statistically significant. The correlation of coagulation and fibrinolysis markers and bleeding volume were analyzed using the Pearson’s correlation coefficient.

## Results

### No differences in bleeding volume or number of transfusion units with or without CPB

Among 48 cases, 42 cases were on CPB. No differences in bleeding volume or number of transfusion units were observed between the cases with CPB and the cases without CPB. There was also no significant difference in bleeding volume among patients with blood type-A, −B, −O and -AB (median value type-A 1402 ml, type-B 1372 ml, type-O 1777 ml, type-AB 884 ml, respectively). The correlation coefficient between dose (unit) of FFP infused and bleeding volume was 0.73.

### Laboratory parameters predicting massive bleeding

**Fig. 1:**
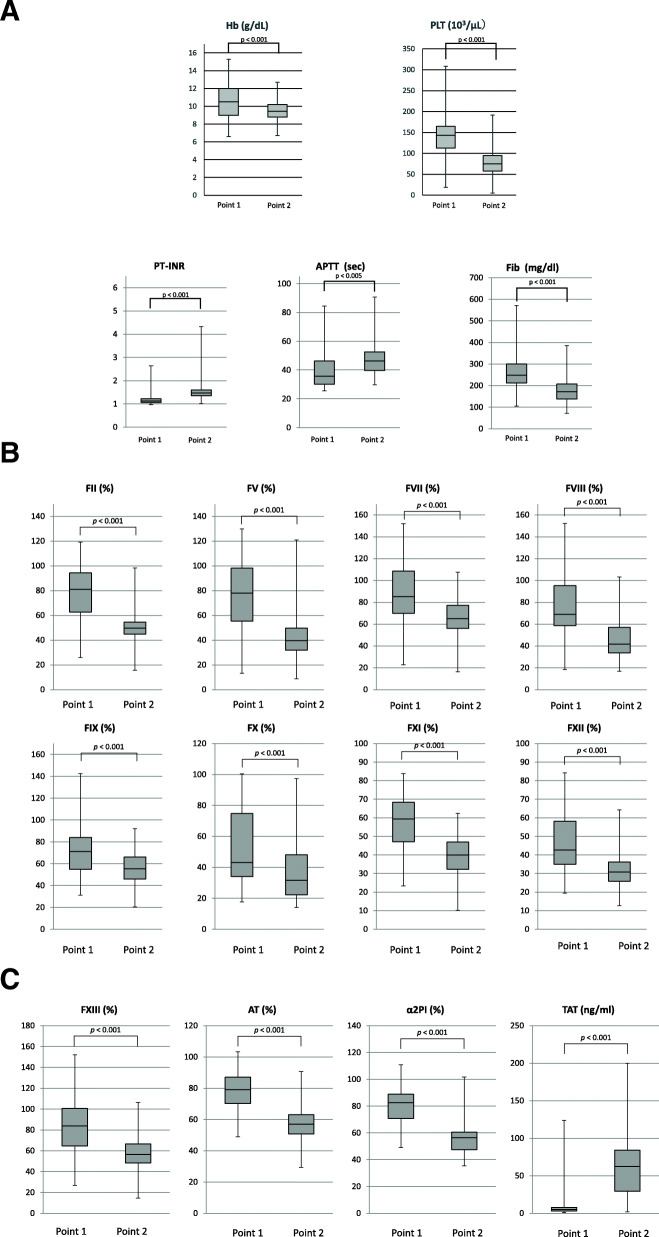
(A) Values of hemoglobin (Hb), platelet (PLT), PT-INR, APTT and fibrinogen (Fib) at point 1 (pre-CPB after induction of anesthesia, or at the time immediately before surgery in patients without CPB) and at point 2 (post-CPB, or at the time right after surgery procedure in patients without CPB). (B) Values of coagulation factor II (FII), FV, FVII, FVIII, FIX, FX, FXI and FXII at point 1 (pre-CPB after induction of anesthesia, or at the time immediately before surgery in patients without CPB) and at point 2 (post-CPB, or at the time right after surgery procedure in patients without CPB). (C) Values of FXIII, antithrombin (AT), α2 plasmin inhibitor (α2PI) and thrombin-antithrombin complex (TAT) at point 1 (pre-CPB after induction of anesthesia, or at the time immediately before surgery in patients without CPB) and at point 2 (post-CPB, or at the time right after surgery procedure in patients without CPB)

**Fig. 2:**
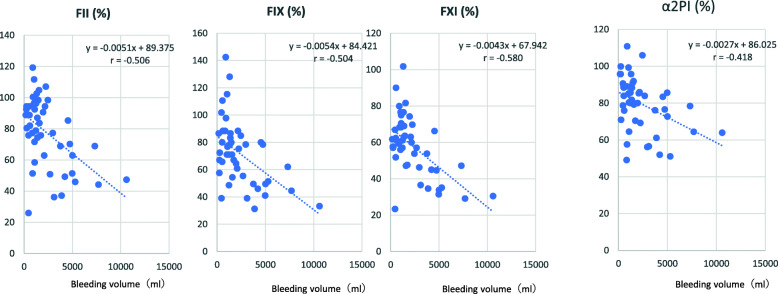
Correlation of bleeding volume and values of FII, FIX, FXI and α2PI at point 1 (pre-CPB after induction of anesthesia, or at the time immediately before surgery in patients without CPB)

**Fig. 3:**
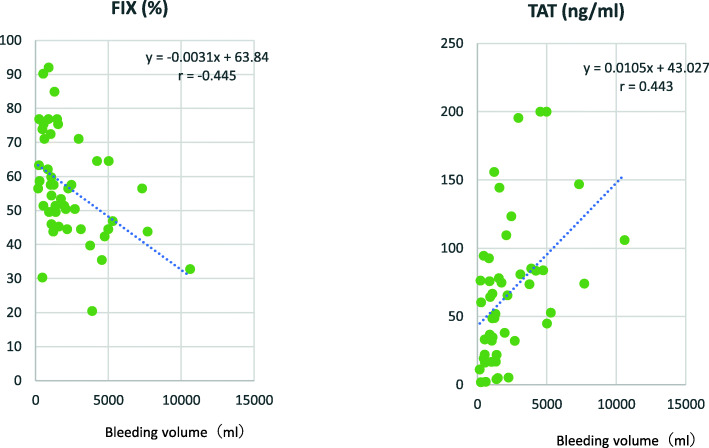
Correlation of bleeding volume and values of FIX and TAT at point 2 (post-CPB, or at the time right after surgery procedure in patients without CPB)

When the laboratory assay values obtained at point 1 were compared with values obtained at point 2, values of hemoglobin, platelet, PT-INR, APTT and Fib at point 2 were changed (Fig. 1A). Activities of coagulation factors (Fig. 1B) and values of FXIII, ATIII and α2PI were also decreased and values of TAT were increased at point 2 (Fig. 1C). Values of FII, FIX, FXI andα2PI at point 1 were negatively correlated with bleeding volume (FII, *r =* − 0.506: FIX, *r =* − 0.504: FXI *r =* − 0.580; α2PI, *r =* − 0.418) (Fig. 2). Correlations between the assay values at point 2 and bleeding volume are shown in Fig. 3. Values of FIX were negatively correlated (*r =* − 0.445) and values of TAT were positively correlated with bleeding volume (*r =* 0.443).

## Discussion

To evaluate the potential predictive value of hemostatic laboratory tests for postoperative bleeding in cardiovascular surgery laboratory markers of coagulation and fibrinolysis were measured and their correlations with bleeding volume were determined. Levels of coagulation factors were uniformly decreased at point 2 as compared to the values at point 1, suggesting that use of anticoagulants and consumption due to operative stress can decrease the levels of coagulation factors. Markers of increased fibrinolysis, such as fibrin-fibrinogen degradation products, D-dimer and plasmin-α2 plasmin inhibitor complex exhibited relatively more volatility than coagulation factors (results not shown). Increase in TAT at point 2 may suggest tissue damage due to operative stress.

Activities of FII, FIX and FXI at point 1 were negatively correlated with bleeding volume. These activities were not below the critical levels necessary for hemostasis even at point 2, suggesting that coagulopathy is not due to a decrease in one single factor but due to the decrease in multiple coagulation factors. Furthermore, ATIII with anticoagulant activity, FXIII with thrombus stabilizing activity andα2PI, hypofibrinolytic marker, were negatively correlated with bleeding volume. In particular, α2PI was closely correlated with bleeding volume. At point 2 TAT was positively correlated with bleeding volume, suggesting that TAT reflected operative stress. Taken together, coagulopathy seen during cardiovascular surgery reflects combined effects of decrease in coagulation factors and factors related to thrombus formation.

This is a single center observational study. The number of patients studied was relatively low and did not allow us to conduct multivariate analysis. The relatively low ability of laboratory markers to predict the volume of postoperative bleeding might be due to the differences in the in vitro testing of coagulation and fibrinolysis and the local hemostatic environment. There might be a local high fibrinolytic activity in the traumatized tissue that is not represented by circulating blood samples [6]. Fibrinogen levels were reported to be moderately associated with postoperative bleeding [7]. Fibrinogen is a key molecule for hemostasis and is relatively easily administered using fibrinogen concentrate [8]. Preoperative fibrinogen administration might reduce postoperative bleeding [9]. Thromboelastometry is also reported to predict major bleeding in pediatric cardiac surgery [10].

There was no increased blood loss in patients with particular blood group. This observation in cardiovascular surgery was in contrast to the previous reports in postpartum blood loss [11, 12]. Prospective further studies taking into account coagulation profile are necessary to determine whether ABO system can be considered a heritable risk of perioperative period hemorrhage.

This is a single center study with relatively small number of patients and the antigen levels of coagulation factors were not measured. Further study involving more patients would clarify factor(s) independently predicting postoperative bleeding.

## Conclusions

Our results suggested that FII, FIX, FXI, FXIII and α2PI are associated with postoperative bleeding volume. Diluted coagulopathy, decrease in stabilized fibrin due to decrease in FXIII and hyperfibrinolysis due to decrease in α2PI may contribute to hemostatic defect. Thrombin burst due to operative stress as represented by increase in TAT values may also contribute to hemostatic defect. Further larger scale clinical studies will improve our understanding of the pathogenesis of coagulopathy and provide good predicting markers of bleeding.

## Data Availability

The datasets used and/or analyzed during the current study are available from the corresponding author on reasonable request.

## Abbreviations

CPB: Cardiopulmonary bypass
RBC: Red blood cells
FFP: Fresh frozen plasma
PC: Platelet concentrate
CABG: Coronary artery bypass grafting
AT: Antithrombin
α2PI: α2 plasmin inhibitor
TAT: Thrombin-antithrombin complex
